# A novel mutation in early‐onset sarcoidosis/Blau syndrome: an association with *Propionibacterium acnes*

**DOI:** 10.1186/s12969-021-00505-5

**Published:** 2021-02-18

**Authors:** Fumiko Okazaki, Hiroyuki Wakiguchi, Yuno Korenaga, Tamaki Nakamura, Hiroki Yasudo, Shohei Uchi, Ryoji Yanai, Nobuyuki Asano, Yoshinobu Hoshii, Tsuyoshi Tanabe, Kazushi Izawa, Yoshitaka Honda, Ryuta Nishikomori, Keisuke Uchida, Yoshinobu Eishi, Shouichi Ohga, Shunji Hasegawa

**Affiliations:** 1 Department of Pediatrics, Yamaguchi University Graduate School of Medicine, 1-1-1 Minamikogushi, 755-8505 Ube, Yamaguchi Japan; 2 Department of Ophthalmology, Yamaguchi University Graduate School of Medicine, Ube, Japan; 3 Department of Dermatology, Yamaguchi University Graduate School of Medicine, Ube, Japan; 4 Department of Diagnostic Pathology, Yamaguchi University Graduate School of Medicine, Ube, Japan; 5 Department of Public Health and Preventive Medicine, Yamaguchi University Graduate School of Medicine, Ube, Japan; 6 Department of Pediatrics, Kyoto University Graduate School of Medicine, Kyoto, Japan; 7 Department of Pediatrics and Child Health, Kurume University School of Medicine, Kurume, Japan; 8 Department of Human Pathology, Tokyo Medical and Dental University Graduate School, Tokyo, Japan; 9 Department of Pediatrics, Graduate School of Medical Science, Kyushu University, Fukuoka, Japan

**Keywords:** D512V mutation, Granulomatous disease, Methotrexate, NF-κB, *NOD2*, *Cutibacterium acnes*, PAB antibody

## Abstract

**Background:**

Early-onset sarcoidosis (EOS) and Blau syndrome (BS) are systemic inflammatory granulomatous diseases without visible pulmonary involvement, and are distinguishable from their sporadic and familial forms. The diseases are characterized by a triad of skin rashes, symmetrical polyarthritis, and recurrent uveitis. The most common morbidity is ocular involvement, which is usually refractory to conventional treatment. A gain-of-function mutation in the nucleotide-binding oligomerization domain-containing protein 2 (*NOD2*) gene has been demonstrated in this disease; however, little is known about the relationship between the activation of *NOD2* and the pathophysiology of EOS/BS. Here we describe EOS/BS with a novel mutation in the *NOD2* gene, as well as detection of *Propionibacterium acnes* (*P. acnes*) in the granulomatous inflammation.

**Case presentation:**

An 8-year-old Japanese girl presented with refractory bilateral granulomatous panuveitis. Although no joint involvement was evident, she exhibited skin lesions on her legs; a skin biopsy revealed granulomatous dermatitis, and *P. acnes* was detected within the sarcoid granulomas by immunohistochemistry with *P. acnes*-specific monoclonal (PAB) antibody. Genetic analyses revealed that the patient had a *NOD2* heterozygous D512V mutation that was novel and not present in either of her parents. The mutant *NOD2* showed a similar activation pattern to EOS/BS, thus confirming her diagnosis. After starting oral prednisolone treatment, she experienced an anterior vitreous opacity relapse despite gradual prednisolone tapering; oral methotrexate was subsequently administered, and the patient responded positively.

**Conclusions:**

We presented a case of EOS/BS with a novel D512V mutation in the *NOD2* gene. In refractory granulomatous panuveitis cases without any joint involvement, EOS/BS should be considered as a differential diagnosis; genetic analyses would lead to a definite diagnosis. Moreover, this is the first report of *P. acnes* demonstrated in granulomas of EOS/BS. Since intracellular *P. acnes* activates nuclear factor-kappa B in a *NOD2-*dependent manner, we hypothesized that the mechanism of granuloma formation in EOS/BS may be the result of *NOD2* activity in the presence of the ligand muramyl dipeptide, which is a component of *P. acnes.* These results indicate that recognition of *P. acnes* through mutant *NOD2* is the etiology in this patient with EOS/BS.

## Background

Sarcoidosis is a multiorgan inflammatory disease with unknown etiology, characterized by the histological features of noncaseating epithelioid cell granulomas [1]. Two distinct types of sarcoidosis have been reported in children: classic sarcoidosis (CS) and early-onset sarcoidosis (EOS) [1, 2]. The former, clinically characterized by a triad of lung, lymph node, and eye involvement, similar to its manifestation in adults, presents in older children and is detectable via chest radiography. The latter is quite rare and found in younger children, presenting with a triad of skin, joint, and eye disorders with no apparent pulmonary involvement.

EOS and Blau syndrome (BS) are systemic inflammatory granulomatous diseases [1, 3]. The pathophysiology is the same for both diseases; however, differentiation between the sporadic and familial (autosomal dominant inheritance) forms is possible [3]. Clinically, in both diseases, the cutaneous and articular symptoms primarily appear in children aged < 4 years [4–6], while ocular manifestations typically appear between the ages of 7 and 12 years [7]. On the other hand, the most common morbidity associated with EOS/BS is ocular involvement that is usually refractory to conventional treatment, including continuous local and systemic glucocorticoids [8].

In 2001, *NOD2* was identified on chromosome 16q12 as the gene responsible for EOS/BS [1, 3, 7, 9]. The *NOD2* gene is intracellularly expressed in phagocytic cells and recognizes muramyl dipeptide (MDP), a component of the bacterial peptidoglycan, inducing an immune response through nuclear factor-kappa B (NF-κB) activation. *NOD2* mutations in patients with EOS/BS enhance the self-oligomerization of *NOD2*, leading to augmented *NOD2* activity even in the absence of the ligand MDP; further increases in activity by the addition of ligands represent gain-of-function mutations, consistent with the dominant mode of inheritance of the granulomatous disease [10, 11]. However, genetic screening of the *NOD2* gene revealed no common mutations in Japanese and Caucasian CS patients [12, 13].

Despite a number of relevant studies, little is known about the relationship between the activation of *NOD2* and the pathophysiology of EOS/BS [14]. The etiologic aspect of CS as an allergic endogenous infection has recently been elucidated; CS is most likely the result of a complex interaction between infection, immunity, and allergic reaction. Sarcoid granulomas form in patients with a hypersensitive immune response to *Propionibacterium acnes* (*P. acnes*). They are formed primarily as a host defense mechanism against intracellular *P. acnes* activated at the sites of latent infection to prevent the spread of the infectious agent [15].

Conversely, no reports have described the association between EOS/BS and *P. acnes.* Contrary to the asymptomatic and sometimes naturally resolving form of the disease found in CS, EOS/BS is progressive and often leads to poor prognoses, such as blindness or joint destruction [16]. Clarifying the pathogenesis of EOS/BS is essential for preventing a poor prognosis. Considering all these factors, we describe here a case of EOS/BS with a novel mutation in the *NOD2* gene as well as the presence of *P. acnes* in the granulomatous inflammation of the biopsied skin tissue.

## Case presentation

During early infancy, a Japanese girl developed papules on her legs and the dorsum of her hands. While the condition remained undiagnosed, the papules on her hand disappeared spontaneously at the age of 4 years. At age 5, she developed both conjunctival hyperemia and photophobia in her left eye; she was diagnosed with anterior uveitis, which was effectively treated using localized therapy. At age 7.5, she developed central scotoma and sudden visual loss (left > right) that was diagnosed as granulomatous panuveitis, which was effectively treated using local glucocorticoid therapy.

**Fig. 1:**
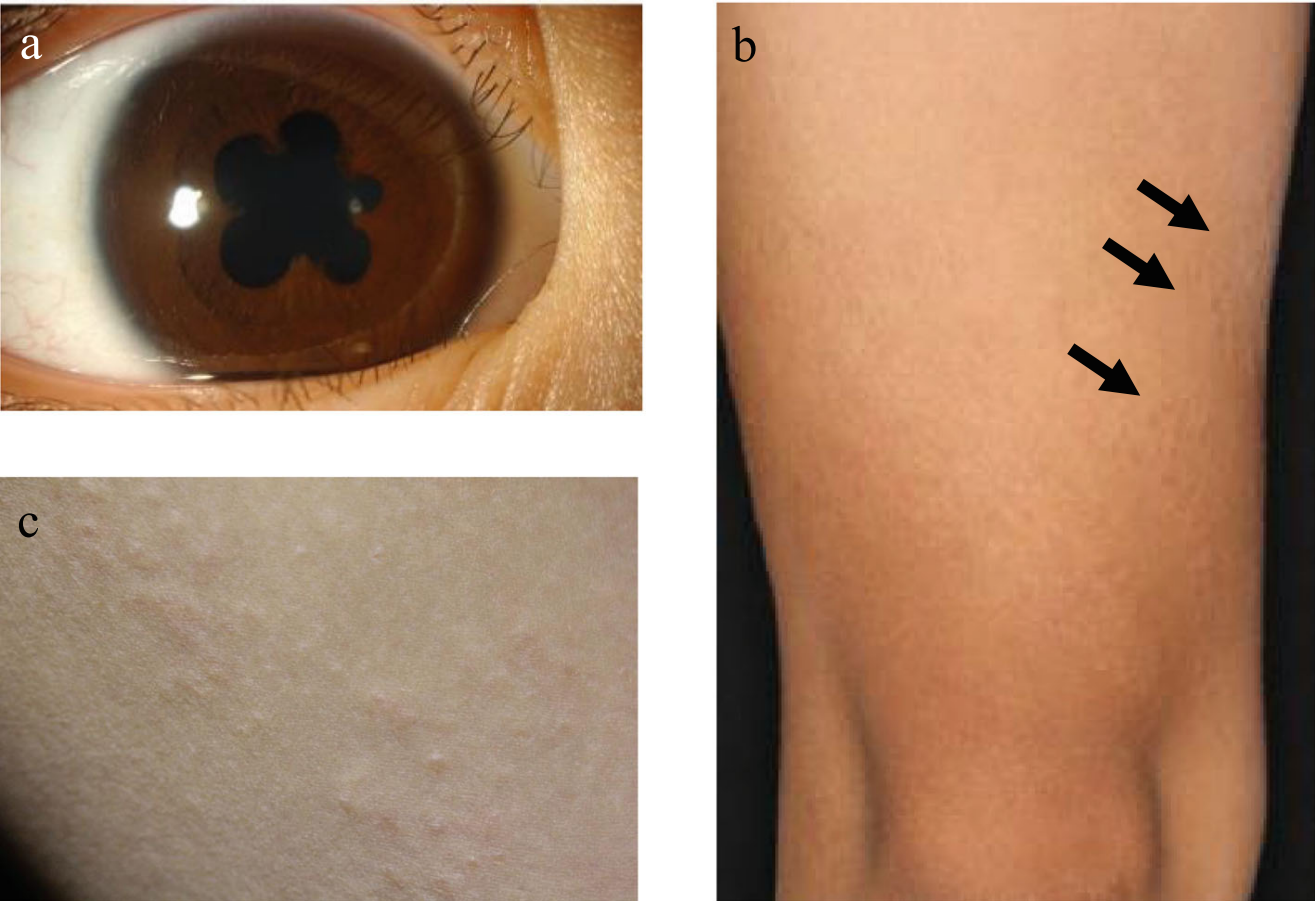
Clinical findings. **a** Anterior chamber inflammation in both eyes and mydriasis due to posterior synechiae in the right eye. **b** and **c** Asymptomatic diffuse papules on the extensor surfaces of the legs (arrows)

**Fig. 2:**
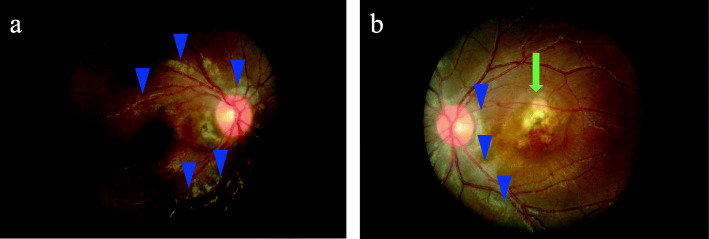
Fundus photograph. Right (**a**) and left (**b**) eyes showing bilateral multifocal choroiditis (arrowheads) presenting as creamy yellow lesions and a scar (arrow) in the macular region of the left eye

By age 8, the granulomatous panuveitis had flared up again; anterior chamber inflammation was seen in both eyes, and mydriasis had developed in the right eye due to posterior synechiae (Fig. 1a). The fundus was characterized by bilateral multifocal choroiditis presenting as creamy yellow lesions, and a scar was seen in the macular region of her left eye (Fig. 2). Systemic glucocorticoid therapy was therefore needed to preserve sight in the left eye. Laboratory tests showed hemoglobin level (11.9 g/dL), white blood cell count (6.22 × 10^9^ /L), platelet count (292 × 10^9^ /L), erythrocyte sedimentation rate (5 mm/h; reference range [RR], 5–15 mm/h), C-reactive protein level (0.01 mg/dL; RR, < 0.27 mg/dL), and matrix metalloproteinase-3 (16.4 ng/mL; RR, < 20.0 ng/mL); the tests for antinuclear antibodies and rheumatoid factor were negative. Her immunological tests were normal, and there were no episodes of recurrent or severe bacterial or fungal infection. Therefore, no additional tests, such as a flow cytometric dihydrorhodamine neutrophil respiratory burst assay, were performed to rule out chronic granulomatous disease. Magnetic resonance imaging of the extremities was carried out to evaluate the presence of arthritis; no abnormalities were detected.

**Fig. 3:**
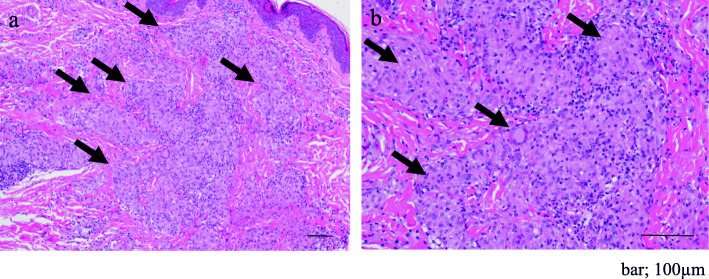
Histopathologic findings. **a** Several noncaseating epithelioid cell granulomas scattered throughout the dermis and subcutaneous tissue (arrows). **b** The epithelioid cell granulomas poorly infiltrated by lymphocytes (arrows). Hematoxylin and eosin stain. Scale bar; 100 µm

**Fig. 4:**
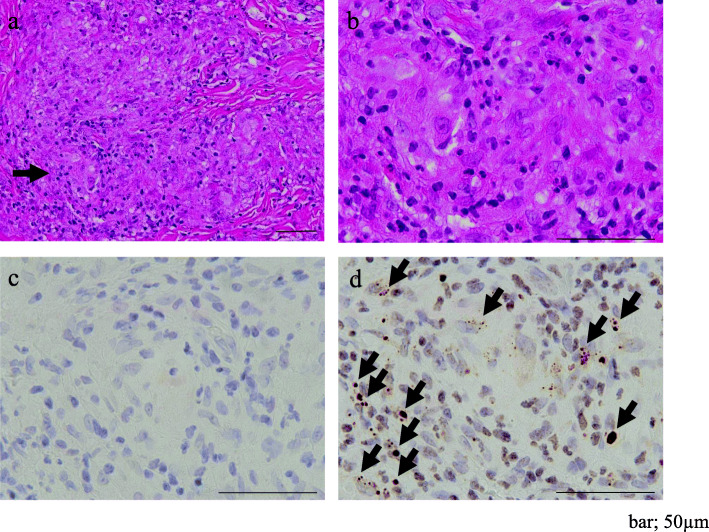
*P. acnes* detected by PAB antibody within the sarcoid granulomas of biopsied skin lesion. Hematoxylin and eosin stain and IHC with PAB antibody and LAM antibody as control are shown pairwise. **a** Noncaseating epithelioid cell granulomas in the dermis. **b** Higher magnification of the granuloma indicated by an arrow in (**a**). **c** No positive signal detected by LAM antibody in the identical granuloma. **d** Many round bodies detected by PAB antibody in the granuloma (arrows). Scale bar; 50 µm. IHC, immunohistochemistry; PAB, *Propionibacterium acnes*-specific monoclonal; LAM, mycobacteria-specific monoclonal

**Fig. 5:**
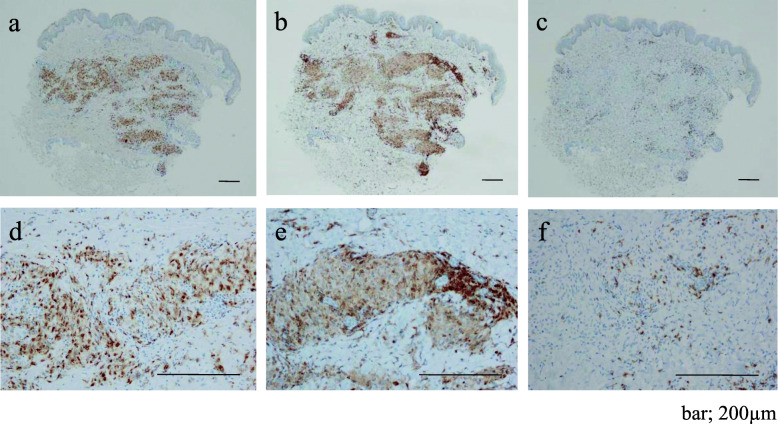
Immunohistochemical findings of CD4^+^, CD8^+^, and CD68^+^ cells. Large granulomas in the dermis and subcutaneous tissue were comprised of CD4^+^ cells (**a** and **d**), and CD68^+^ cells (**b** and **e**). CD8^+^ cells were rarely detected (**c** and **f**). Scale bar; 200 µm

A skin biopsy from papules on her leg indicated granulomatous dermatitis (Figs. 1b and c and 3). The biopsy samples were examined by immunohistochemistry (IHC) with a *P. acnes*-specific monoclonal antibody (PAB antibody, D371-3, MEDICAL & BIOLOGICAL LABORATORIES CO., LTD., Aichi, Japan) and a mycobacteria-specific monoclonal antibody (LAM antibody, D372-3, MEDICAL & BIOLOGICAL LABORATORIES CO., LTD.), according to the methods described in the original study [17]. The PAB antibody detected several signals of round bodies within the sarcoid granulomas and in lesions infiltrated by inflammatory cells, while no positive signal was observed using the LAM antibody as control (Fig. 4). The sarcoid granulomas were comprised of CD4^+^ cells and CD68^+^ cells; CD8^+^ cells were rarely observed in the dermis and subcutaneous tissue (Fig. 5).

**Fig. 6:**
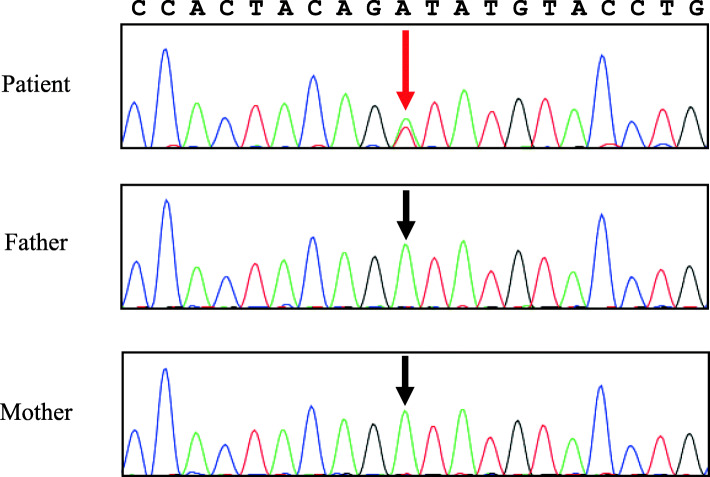
*NOD2* heterozygous mutation. The electropherogram shows the sequence of heterozygous c.1535A > T transition in exon 4 of the nucleotide-binding oligomerization domain in the patient (red arrow). No such mutation was found in her parents (black arrows). *NOD2*, nucleotide-binding oligomerization domain-containing protein 2

Genetic analyses were performed of both the patient’s and her parents’ *NOD2* genes. Genomic DNA was extracted from the peripheral blood of the patient and her parents, and all 12 exons of the *NOD2* gene, including exon-intron boundaries, were amplified by polymerase chain reaction and sequenced by Sanger method. Results revealed that the patient had a heterozygous c.1535A > T mutation on exon 4 in the nucleotide-binding domain of *NOD2*, while her parents did not. Analysis of the protein variant revealed that the mutation was D512V (p.Asp512Val) (Fig. 6). An NF-κB luciferase assay was performed. HEK293T cells (1 × 10^5^) were cotransfected with 30 ng of the expression construct of the *NOD2* variant together with an NF-κB reporter plasmid. NF-κB activity was measured after 12 h of incubation either with or without 5 µg/mL MDP. A mock vector and the wild-type *NOD2* were used as controls [1]; R334W and H496L were used as positive controls [1]. An in vitro assessment of the mutation indicated an increased basal NF-κB activity that increased further after MDP stimulation [18], demonstrating a strong association with EOS/BS.

Based on her clinical, histological, and genetic features, the patient was diagnosed with EOS/BS despite the absence of arthritis during the course of the disease. She was treated with oral prednisolone (PSL) (1.5 mg/kg/day). Six months after initiating treatment, she experienced a relapse of anterior vitreous opacity while on a 0.3 mg/kg/day dose of oral PSL, which was then gradually tapered. Oral methotrexate (10 mg/m^2^/week) was prescribed as an additional treatment, to which there was a positive response. She has remained in clinical remission for over three years on a 0.1 mg/kg/day dose of oral PSL with methotrexate.

## Discussion

This report describes a case of EOS/BS with a novel D512V mutation in the *NOD2* gene and a lack of arthritis. *P. acnes* was also detected in the granulomatous inflammation, which leads to the elucidation of the etiology of EOS/BS.

Our patient did not show any symptoms of arthritis. However, the most common manifestation of EOS/BS is symmetric and usually painless polyarthritis [19]. Only three mutations, E383K, R587C, and R334W, were identified previously in EOS/BS patients without arthritis [18, 20]. The R334W mutation in the *NOD2* gene is one of the most frequently occurring mutations in patients with EOS/BS [18, 20]. The patient with R587C mutation was a familial case with an age of onset of 216 months, and demonstrated skin rash without arthritis and uveitis [20]. Her daughter, who had the same mutation, was the proband of the disease and had rash, uveitis, and arthritis with an age of 21 months at onset [20]. It may be difficult to identify phenotypic variations, such as the timing and site of onset and the clinical severity of EOS/BS, according to the *NOD2* genotype. Nongenetic factors such as environmental conditions and/or infectious agents might be involved in phenotypic variation and clinical severity. Therefore, in individuals with *NOD2* mutations, *P. acnes* might be the trigger for onset of the disease and may determine the onset site by causing intracellular proliferation from a latently infected state. In our patient, translocation of *P. acnes* after local proliferation at the early onset site of the skin may have caused latent infection only in the eye (but not in the joint), where later reactivation caused uveitis (but not arthritis).

Our patient had a novel D512V mutation of the *NOD2* gene. Functional analysis showed elevated background activity and augmented activity after ligand stimulation, demonstrating a strong association with EOS/BS. A recent report elucidated the etiological aspect of CS as an allergic endogenous infection caused by *P. acnes* [15]. Histological localization of *P. acnes* within sarcoid granulomas of the lymph nodes has been demonstrated by *in situ* hybridization [21] and IHC with PAB antibodies [17], suggesting that *P. acnes* is related to the cause of granuloma formation in CS. *P. acnes* has also been found in the cutaneous sarcoid granulomas of CS patients by IHC with PAB antibodies [22–26]. PAB antibody is a *P. acnes*-specific monoclonal antibody that reacts with lipoteichoic acids that are membrane-anchored molecules in the cell envelopes of gram-positive bacteria. In our case, using the same PAB antibody used in the previous studies, IHC detected several round bodies of *P. acnes* both within and around the sarcoid granulomas. The granulomas were comprised of CD4^+^ and CD68^+^ cells, indicating that they formed by the accumulation of CD4^+^ T-lymphocytes and macrophages. Therefore, the granulomas may have developed as a Th1 immune response to *P. acnes*.

*P. acnes* has also been demonstrated in ocular CS lesions. Nagata et al. [27] reported that *P. acnes* was present within granulomas in 10 (83 %) of 12 retinal biopsy samples from 9 (82 %) of 11 patients with ocular CS, whereas it was not detected in any (0 %) of the control group; the bacteria were identified as round bodies that reacted with PAB antibody. The retina is an aseptic environment and the presence of *P. acnes* within the retina is extremely unusual; therefore, detection of *P. acnes* within the retina was a critical observation, confirming the role of *P. acnes* as an etiological agent for CS. *P. acnes* is commensal on the skin, including sebaceous follicles, but usually absent in the dermis; therefore, we considered that detection of *P. acnes* within the sarcoid granulomas in the dermis strongly suggested *P. acnes* as an etiological agent for EOS/BS.

Tanabe et al. [28] found that both *NOD1* and *NOD2* proteins recognized intracellular *P. acnes*. Furthermore, systematic searches for *NOD1* gene polymorphisms in Japanese CS patients identified an increased frequency of G796A. Functional analysis revealed that *NOD1* G796A was associated with a lower expression at the protein level, leading to reduced NF-κB activation in response to intracellular *P. acnes*. These results indicated that impaired recognition of intracellular *P. acnes* through *NOD1* variants might cause susceptibility to CS within the Japanese population. We, therefore, hypothesized that the mechanism of granuloma formation in EOS/BS may be the result of *NOD2* hyperactivity in the presence of the ligand MDP, a component of *P. acnes*.

We found that EOS/BS and CS share a common feature of *P. acnes* in granulomatous pathology; however, the reason for the differing clinical features (especially the different triad of symptoms) between EOS/BS and CS remains unknown. It may be related to the presence or absence of *NOD2* mutations, or different sites of preceding latent infection of *P. acnes* in EOS/BS and CS. The lack of arthritis in our patient may indicate the lack of latent infection. Additionally, it remains unknown how the hyperactivity of NF-κB without MDP stimulation is involved in the pathophysiology of EOS/BS. The gain-of-function *NOD2* mutations found in EOS/BS patients may cause an excessive immune response to *P. acnes*, with increased *NOD2* activity in the presence of MDP; this excessive immune response to *P. acnes* would lead to poor prognosis.

Nagakura et al. [29] reported that tumor necrosis factor inhibitors provide long-term clinical benefits in EOS/BS. Thus, it would be one of the candidate treatments in the case of a later relapse. Furthermore, according to the identification of *P. acnes* in our patient, a combination of antibiotic and symptomatic therapy might be considered as a novel treatment for EOS patients. Antibiotics against *P. acnes* may be effective for patients with progressive CS by preventing relapses of inflammation caused by repeated reactivation of the latent bacteria [15]. The result of a nationwide questionnaire survey, performed by a Japanese research group in 2005, indicated that antibiotic therapy was effective in 43 % of patients with CS treated with several kinds of antibiotics, including minocycline, doxycycline, and clarithromycin [15]. This study therefore provides scope for future research.

## Conclusions

In summary, we presented a case of EOS/BS with a novel D512V mutation in the *NOD2* gene. In refractory granulomatous panuveitis cases without joint involvement, EOS/BS should be considered as a differential diagnosis; genetic analyses would lead to a definite diagnosis. Moreover, this is the first report of *P. acnes* demonstrated in granulomas of EOS/BS. Since intracellular *P. acnes* activates NF-κB in a *NOD2-*dependent manner, we hypothesized that the mechanism of granuloma formation in EOS/BS may be the result of *NOD2* activity in the presence of the ligand MDP, which is a component of *P. acnes.* These results indicate that recognition of *P. acnes* through mutant *NOD2* is the etiology in this patient with EOS/BS.

### Additional information

This patient is the same as the D512V case reported in reference number 18. There was no polyarthritis or renal calcification in this patient. In fact, the statement that polyarthritis and renal calcification were present is an error made by the authors of reference number 18, and is currently being corrected by them for submission of an erratum.

## Data Availability

Not applicable.

## Abbreviations

BS: Blau syndrome
CS: Classic sarcoidosis
EOS: Early-onset sarcoidosis
IHC: Immunohistochemistry
LAM: Mycobacteria-specific monoclonal
MDP: Muramyl dipeptide
NF-κB: Nuclear factor-kappa B
*NOD2*: Nucleotide-binding oligomerization domain-containing protein 2
PAB: *Propionibacterium acnes*-specific monoclonal
*P. acnes*: *Propionibacterium acnes*
PSL: Prednisolone
RR: Reference range
